# Multimodal diagnostic imaging in primary vitreoretinal lymphoma

**DOI:** 10.1186/s40942-022-00405-0

**Published:** 2022-08-26

**Authors:** Lucy T. Xu, Ye Huang, Albert Liao, Casey L. Anthony, Alfredo Voloschin, Steven Yeh

**Affiliations:** 1 Retina Group of Washington, Washington, DC USA; 2 Truhlsen Eye Institute, University of Nebraska Medical Center, Omaha, NE USA; 3 Emory Eye Center, Emory University School of Medicine, Atlanta, GA USA; 4 Department of Hematology and Medical Oncology, Emory University School of Medicine, Atlanta, GA USA; 5 Winship Cancer Institute, Emory University School of Medicine, Atlanta, GA USA

**Keywords:** Primary vitreoretinal lymphoma, Multimodal imaging, Fundus photography, Fundus autofluorescence, Optical coherence tomography, Fluorescein angiography, Indocyanine green angiography, Electroretinography

## Abstract

**Background:**

Primary vitreoretinal lymphoma (PVRL) is an aggressive lymphoma that may present with protean features and represents a diagnostic challenge. Given that patients with PVRL are at high risk of CNS involvement with a high mortality and morbidity rate, prompt diagnosis is crucial to initiate treatment early in the disease course. A multimodality imaging approach including fundus photography, fundus autofluorescence (FAF), optical coherence tomography (OCT), fluorescein and indocyanine angiography, and electroretinography (ERG) can provide information to establish a diagnosis and provide objective measures for management. We review key findings seen via these imaging modalities in patients with PVRL.

**Observations:**

Fundus photography can highlight commonly seen patterns of PVRL including vitritis, subretinal disease, retinal pigment epithelial (RPE) abnormalities, optic nerve edema, retinal detachment, and less typical retinitis-like lesions. FAF can identify characteristic patterns of hyper- and hypoautofluorescent signal abnormalities in the macula. Spectral-domain OCT will demonstrate vitreous cells, RPE nodularity, and hyperreflectivity of the outer retina. The presence of a hyper-reflective band in the subretinal space and infiltrates between the RPE and Bruch’s membrane can assist in distinguishing PVRL from choroidal lymphoma. Vertical hyperreflective columns (VHRLs) are another pertinent finding that may represent microinfiltrates of the tumor. OCT has proven to be a particularly useful modality in assessing the progress of treatment in PVRL. Fluorescein angiography can show RPE changes, which include granularity, late staining at the RPE level, and blockage. Indocyanine green angiography (ICGA) primarily shows hypocyanescence, which corresponds to PVRL lesions on fundus photography and may occur secondary to loss of RPE and choriocapillaris.

**Conclusion:**

While PVRL remains a challenging disease to diagnose and follow, the use of a multimodality imaging approach may assist in establishing a diagnosis. Because of the anatomic spaces PVRL may affect, fundus photography, OCT, FAF, angiography, and ERG can identify key characteristics of the disease, differentiate PVRL from other diseases, and provide baseline information for targeted systemic and local therapies. Further assessment of anatomic and functional targets will aid our clinical application of multimodal imaging in the management of PVRL.

## Background

Primary vitreoretinal lymphoma (PVRL), also known as primary intraocular lymphoma, is the most common intraocular lymphoma with approximately 380 incident cases in the United States annually [1]. It is an aggressive high-grade lymphoma, usually of the diffuse large B-cell subtype [2]. PVRL is a rare subset of primary central nervous system (CNS) lymphoma. While approximately 15% of patients with primary CNS lymphoma develop PVRL, 65–90% of patients with PVRL develop primary CNS lymphoma [1]. Primary CNS lymphoma is a rare primary CNS tumor comprising only 2% of primary CNS tumors that most frequently presents in the elderly or immunocompromised populations [3]. However, it is associated with high mortality and morbidity. Specifically, the median survival for patients is 10 months, and for those older than 70, the median survival is 6–7 months [4, 5].

PVRL is a well-known masquerade syndrome that may be challenging to diagnose [6]. The mean time from ocular symptoms to diagnosis is 40 months, but the timing from symptom onset to diagnosis has been reported to be up to 12 years in some instances [7]. The delay in PVRL diagnosis can subsequently impede primary CNS lymphoma diagnosis and treatment, impacting survival outcomes for the patient. Thus, an accurate and timely diagnosis of PVRL is imperative. The gold standard of diagnosis is histologic and immunochemical confirmation via a sample obtained by a diagnostic vitrectomy, retinal or subretinal biopsy [1]. However, recent advances in ophthalmic imaging have identified features that support the diagnosis of PVRL. These features can be used to reduce the need for invasive tissue diagnosis in some cases, decrease time to achieve a definitive diagnosis, and provide objective measures to monitor treatment response (see Table 1). In this review, we summarize the multimodal findings on ophthalmic ancillary testing presented in the literature. We review characteristic findings of PVRL found on various imaging modalities, including fundus photography, fundus autofluorescence (FAF), optical coherence tomography (OCT), fluorescein angiography (FA), indocyanine green angiography (ICGA), and electroretinography (ERG).

**Table 1:** Findings on imaging of primary vitreoretinal lymphoma

Imaging modality	Features found on imaging of PVRL	Utility in diagnosis or workup of PVRL
Fundus photography	Visualization of vitreous haze and cellular debris, optic nerve edema, perivascular sheathing, sub-retinal pigment epithelium cream-colored lesions, serous retinal detachments, atypical features mimicking viral retinitis	Direct visualization of findings and monitoring disease progression over time
Fundus autofluorescence (FAF)	Hyperautofluorescence of sub-RPE lesions and alternating or stippled hyper/hypoautofluorescence of the macula	Identification of diffuse and focal areas of RPE disturbance
Optical coherence tomography (OCT)	Vitreous cells, RPE nodularity, outer retinal hyper-reflectivities	Monitor response to treatment by assessing for presence of hyperreflective subretinal lesions (representative of lymphomatous infiltrate)
Fluorescein angiography (FA)	Granularity, late staining at RPE level, blockage; reversed granular pattern of hyperfluoresence and hypofluorescence compared to FAF	May assist in monitoring for progression of disease or recurrence
Indocyanine green angiography (ICGA)	Hypocyanescence	Can help rule out other syndromes with characteristic ICGA findings (i.e. sarcoidosis or white dot syndromes) that can be similar to PVRL
Electroretinography (ERG)	Reduced cone and rod responses; negative wave-form ERG on bright flash response with diminished b-wave response smaller than corresponding a-wave	More research on utility of ERG needed for PVRL

## Fundus Photography (Fig. 1A)

**Fig. 1:**
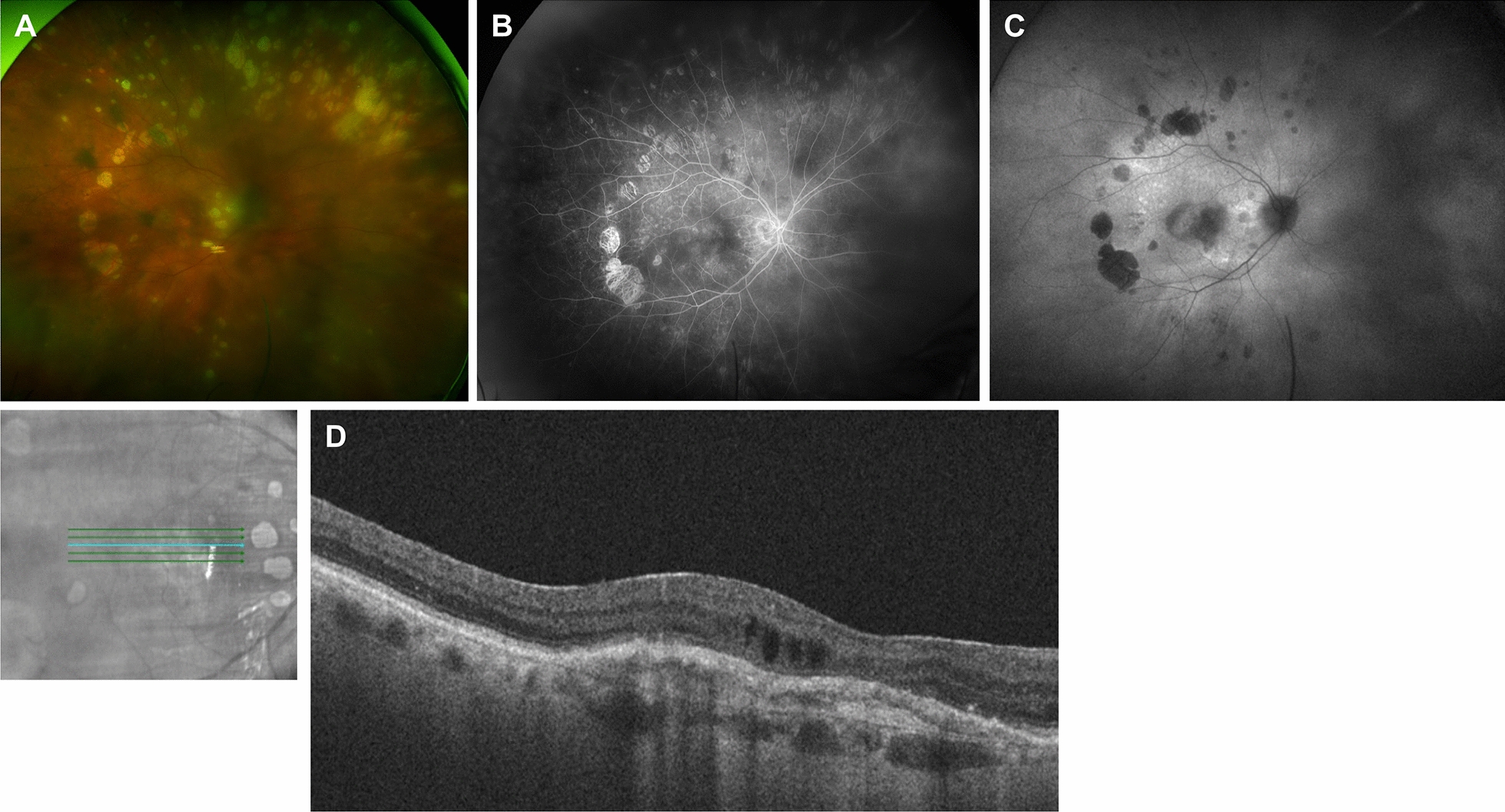
Clinical imaging features of a patient with a diagnosis of PVRL and macular degeneration. **A** Color fundus photograph of the right eye showing vitreous opacity and multiple chorioretinal lesions with variable pigmentation **B** Fluorescein angiography showing staining of chorioretinal lesions and retinal pigment epithelial (RPE) changes. **C** Fundus autofluorescence of the right eye showing corresponding areas of hypoautofluorescence. **D** OCT showing RPE elevation and overlying cystoid edema

Clinical features of PVRL seen on fundus photography include vitreous haze or clumps, optic nerve edema, perivascular sheathing, sub-retinal pigment epithelium (RPE) lesions, cream-colored lesions, or serous retinal detachments [8]. In an evaluation of 43 eyes of 23 patients with PVRL, four patterns were detected on fundus photography: vitritis (46% of eyes), subretinal lesions (46%), optic nerve edema (10%), and retinal detachment (12%) [9]. Specifically, eyes with only vitritis showed visual acuities of 20/27 at 6 months and 20/34 at 12 months post-presentation. Conversely, eyes with optic disc edema or retinal detachment had an average visual acuity of hand motion at 6 months and light perception at 12 months [9]. In 47% of eyes, ultra-widefield fundus photography detected more abnormalities than standard 30 degree images [9]. Fundus photography may also assist in monitoring PVRL changes over time. Mantopoulos and Cebulla report a biopsy-proven case of PVRL with spontaneous regression of a sub-RPE lesion documented on color fundus photographs and OCT [10].

In addition to the typical findings of vitritis, subretinal lesions, and RPE change, fundus photography can also document atypical lesions. For example, PVRL and secondary vitreoretinal lymphoma (VRL) may rarely present with a retinitis-like picture [11–13]. Suspicion for PVRL should be raised if individuals with retinitis-like lesions fail to respond to antiviral therapy, particularly if molecular diagnostics for a viral etiology are negative.

### Fundus autofluorescence

**Fig. 2:**
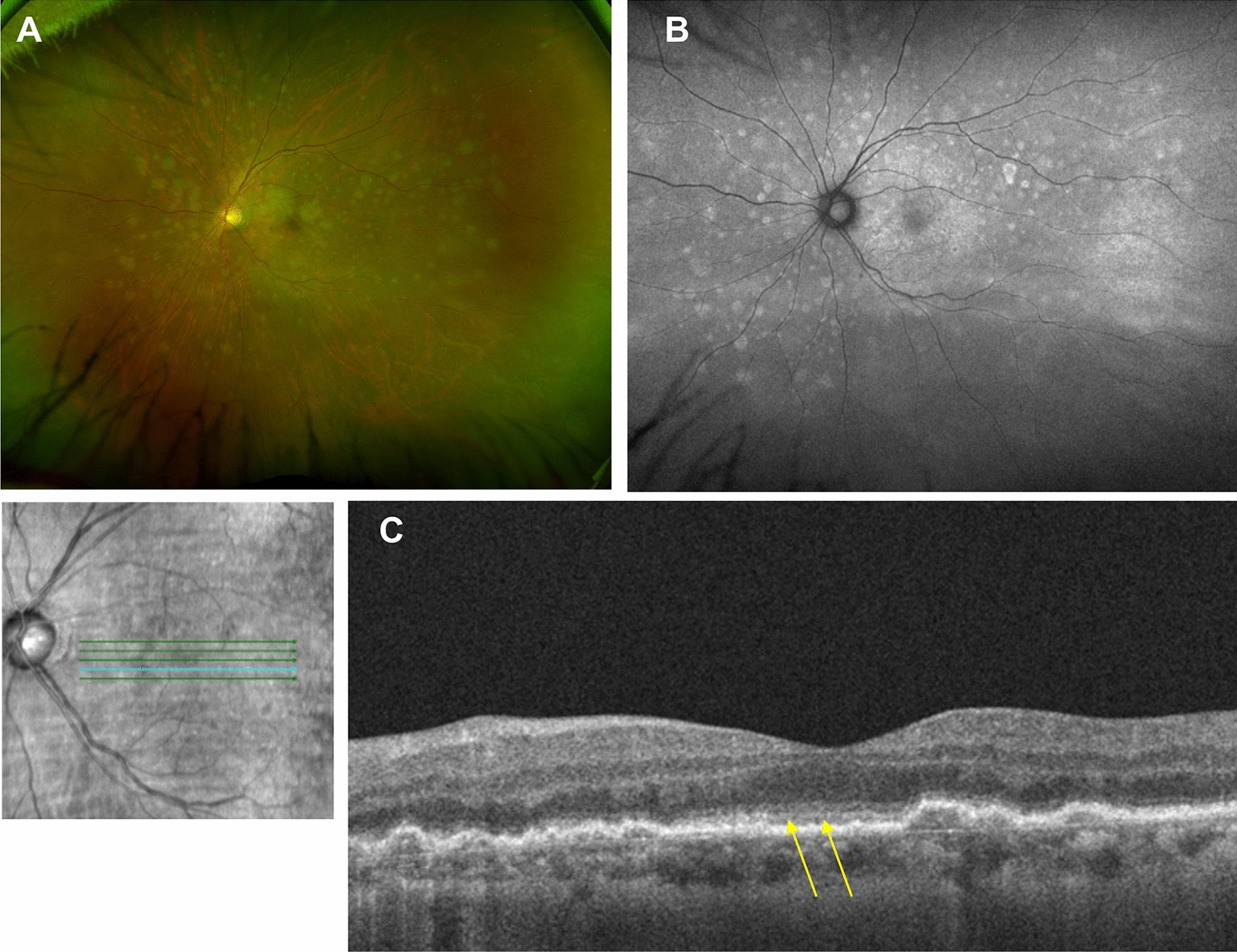
Clinical imaging features of a patient with primary central nervous system lymphoma and recurrent PVRL. **A** Color fundus photograph of the left eye showing multiple hypopigmented lesions. **B** Fundus autofluorescence of the left eye showing corresponding hyperautofluorescent spots within the posterior pole and mid-periphery. **C** OCT showing nodular lesions at the level of the RPE and outer retinal/ellipsoid zone irregularity (yellow arrows)

**Fig. 3:**
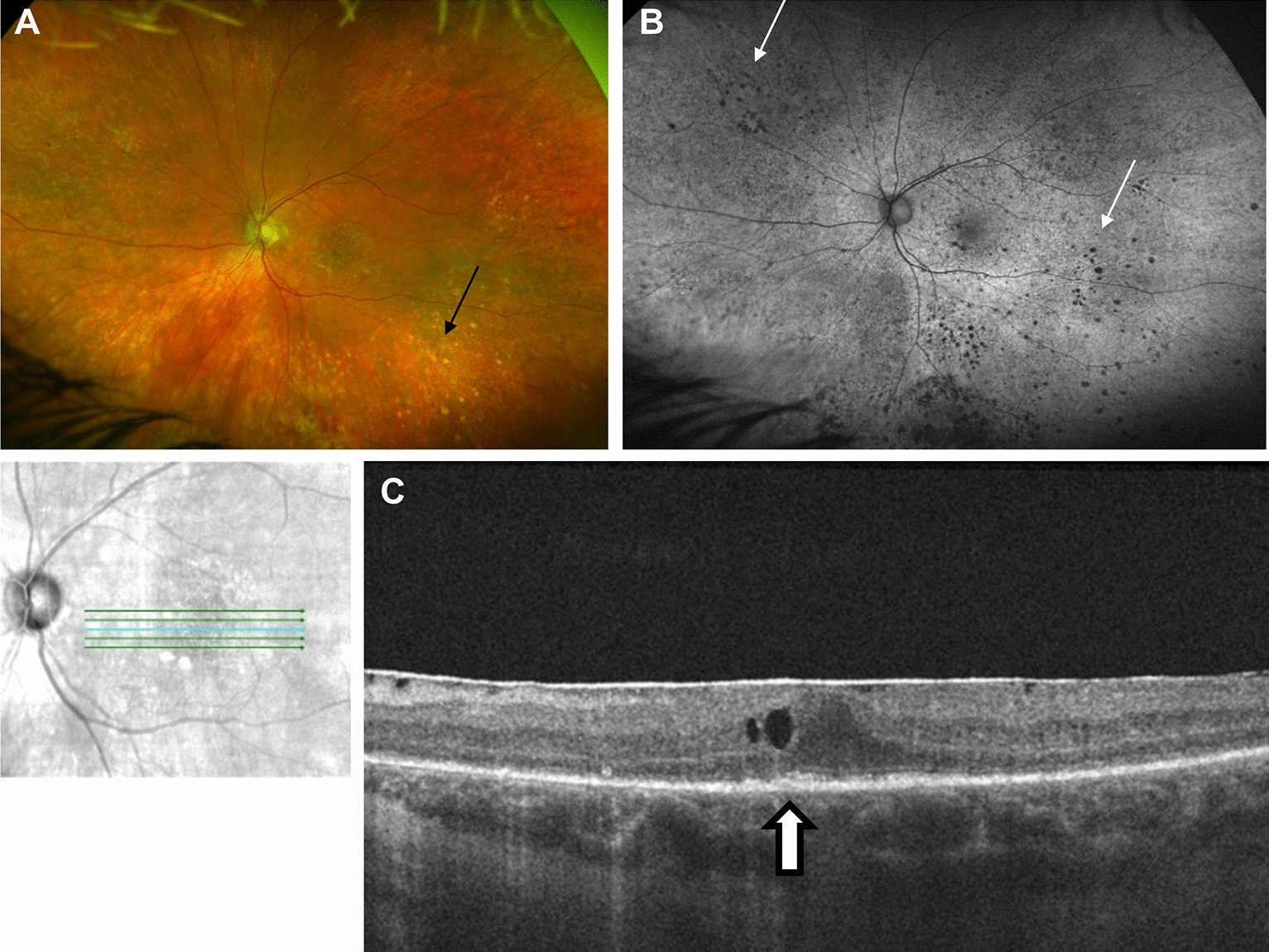
Clinical and imaging features of a patient with a diagnosis of PVRL. **A** Color fundus photograph of the left eye showing discrete yellow chorioretinal lesions (black arrow). **B** Fundus autofluorescence showing small, hypoautofluorescent spots (small white arrows) consistent with PVRL. **C** OCT showing hyperreflective RPE clumping and irregularity (white arrow with black outline) consistent with PVRL

A variety of fundus autofluorescence (FAF) patterns has been reported. During active PVRL, hyperautofluorescence of the sub-RPE lesions and alternating hyper- and hypoautofluorescence of the macula are the most commonly seen patterns [9, 14, 15]. The hyperautofluorescent spots have also been found to correlate with nodular hyperreflective spots on OCT [14]. The granular hyperautofluorescence and hypoautofluorescence pattern on FAF was reversed on fluorescein angiogram [16] (Figs. 1B, C). Egawa et al. hypothesized that lymphomatous RPE infiltration in the form of sub-RPE lesions could alter RPE metabolism and manifest as hyperautofluorecence [16] (Fig. 2B). In areas where there appears to be lymphomatous infiltration above the RPE, the FAF pattern is hypoautofluorescent, possibly due to tumor blockage of normal RPE fluorescence [16, 17] (Fig. 3B). Hypoautofluorescence can also be seen in cases of RPE atrophy after regression of the lesion [14, 15, 17]. Interestingly, these lesions often appear hook-shaped [17]. Hyperautofluorescent dots were also seen in an inactive eye that was previously active [9]. Thus, while FAF patterns can be extremely helpful to identify diffuse and focal areas of RPE disturbance, the variegated pattern makes it challenging to draw conclusions about disease activity based solely on FAF patterns.

Importantly, a normal fundus autofluorescence does not rule out PVRL. In a report by Casady et al., seven of 18 eyes (39%) did not have any remarkable findings on FAF [14]. Of these, two eyes (one patient) were in remission, and five eyes (four patients) were active [14].

### Optical coherence tomography

OCT often demonstrates vitreous cells (100% of eyes), RPE nodularity (63%), and outer retinal hyperreflectivities (43%) [9, 18, 19] (Figs. 2C and 3C). Less common findings included pigment epithelial detachments (PEDs) (30%-50%) (Figs. 1D and 3C), epiretinal membranes (19–36%), and retinal fluid or disorganization (13–17%) [9, 19–21].

Barry et al. describe specific findings that are highly suggestive of PVRL, including a confluent hyper-reflective band in the subretinal space and infiltrates localized between the RPE and Bruch’s membrane. These characteristics can help distinguish PVRL from choroidal lymphoma, which presents with infiltrates deep to Bruch’s [22].

OCT is an excellent tool to monitor response to treatment in PVRL. There are multiple reports of hyperreflective subretinal lesions on OCT that likely represent lymphomatous infiltrate as they resolve or develop into subretinal fibrosis with treatment [19, 20, 23–27]. In one report, after completion of treatment, all abnormalities on initial OCT vanished, including subretinal fluid, outer retinal fuzzy borders, PED, and intraretinal infiltration [19]. However, stigmata of retinal destruction appeared, including subretinal fibrosis, disruption of the outer retina, and retinal thinning [19].

An OCT imaging finding termed “vertical hyperreflective columns” (VHRLs) was found in 58.3% of eyes with vitreoretinal lymphoma [28]. Of note, 2 of the 7 patients had secondary vitreoretinal lymphoma [28]. Hyperreflective intraretinal bands had previously been described in studies; however, these VHRLs extended from the inner retina to the RPE and varied in width [22, 28, 29]. They were commonly located along major vessel arcades or temporally to the fovea but were not detectable on color or infrared photography [28]. VHRLs are thought to represent microinfiltrates of tumor [28].

Anterior segment OCT has been used to diagnose recurrence of PVRL in a previously vitrectomized patient [30]. The patient was symptomatic with decreased vision and was clinically found to have anterior vitreous opacification and cells [30]. The anterior segment OCT demonstrated vitreous debris in the retrolenticular space [30].

### Fluorescein angiography

RPE changes from PVRL are highlighted on FA and are the most common finding. These changes include granularity, late staining at the RPE level, and blockage [31] (Fig. 1B). The granular pattern of hyperfluorescence and hypofluorescence seen on FA is reversed in FAF [16]. FA patterns in PVRL patients often have a “mottled” appearance despite the absence of clinical RPE disturbances. It is hypothesized that diffuse lymphomatous infiltrates under the RPE are impermeable to fluorescein since tumor cell membranes act as a barrier, and the infiltrates would also block choroidal hyperfluorescence [31]. However, some infiltrates behave like drusen and manifest as late staining on FA [31].

PEDs in PVRL patients present with varying patterns on FA. Some demonstrate filling patterns typical for a serous PED: a discrete well-circumscribed area of fluorescence through the transit phase with pooling in the late phase. These findings are thought to result from injured or dead tumor cells with a resultant zone of hyperfluorescence or a serous PED with a sparse collection of tumor cells [31]. PEDs may also be hypofluorescent on FA, likely from a dense collection of viable tumor cells [9, 31].

One prior report described capillary dropout by FA in a patient with biopsy-proven PVRL [32]. The FA demonstrated well-defined areas of capillary dropout across the retina in one eye without disc leakage, cystoid macular edema (CME), or vascular leakage [32]. There was also no evidence of vascular occlusion or findings indicating diabetic retinopathy [32]. The authors hypothesized that the capillary dropout might be due to lymphomatous cells in the inner retinal layer and subsequent retinal vascular occlusion.

Notably, more common evidence of active uveitis, including optic nerve staining/leakage, perivascular staining/leakage, and petaloid leakage of CME, are less prevalent in PVRL patients. Optic nerve leakage/staining was found in 4–45% of patients [7, 31]. Vascular leakage was present in 6–36% of patients [31, 33]. In one study, leakage improved after treatment in all patients and reappeared upon disease recurrence [9]. CME was seen in six eyes (19%) in one study but only in one eye that did not have a prior history of intraocular surgery [31]. Thus, while CME, optic disc leakage, and retinal vascular leakage may be less prevalent than in other cases of endogenous uveitis, the presence of these findings should not exclude lymphoma in the differential diagnosis.

### Indocyanine green angiography

The most commonly observed finding on indocyanine green angiography (ICGA) in PVRL is hypocyanescence. Hypocyanescent lesions were found in 77% of eyes in one study [33]. Another study found that 26% of eyes with PVRL had small round hypocyanescent lesions that erased at the late phase and that 12% of eyes demonstrated large hypocyanescent lesions [23]. Lavine et al. noted the hypocyanescence found in 10 of 12 eyes (83%) in their series correlated with scleral staining [9]. These areas of scleral staining corresponded to white lesions on fundus photos as well as hypoautofluroescence and late hyperfluorescence on FA. This constellation of findings on multi-modal imaging indicates a loss of both RPE and choriocapillaris, making “scleral staining” a more apt term than “window defects” [9]. ICGA was unremarkable in areas of active sub-RPE lesions and active FA leakage [9]. The utility of ICGA in the diagnosis of PVRL is limited due to its inability to demonstrate findings missed by FA or FAF [9]. However, ICGA can help rule out other syndromes with more characteristic ICGA findings, such as sarcoidosis, birdshot retinochoroidopathy, or other white dot syndromes, that often can be difficult to differentiate from PVRL.

### Electroretinography

One case report of a patient with suspected PVRL demonstrated reduced cone and rod responses on full-field electroretinography (ERG) [34]. The patient also had a negative waveform ERG on the bright flash response with a diminished b-wave response that was reduced when compared to its corresponding a-wave [34]. 8 weeks after systemic chemotherapy, rod and cone responses improved but did not fully normalize [34]. However, the negative waveform reverted to normal [34]. By 3 years of follow-up, the amplitudes continued to improve but still remained abnormal [34].

## Conclusion

PVRL can often mimic other ocular diseases, making diagnosis difficult and delayed. Identification of key findings on multimodality ophthalmic imaging modalities can help to identify pathologic lesions that raise the level of suspicion for PVRL, potentially triggering a definitive diagnostic procedure. Characteristic findings include vitritis and subretinal or sub-RPE lesions that may represent tumor infiltrates. Sub-RPE lesions can be hypo- or hyperautofluorescent and do not always correlate with disease activity. Granular fundus autofluorescent changes commonly present with a reversed pattern on FA.

OCT can be used to assess disease activity. OCT can demonstrate a confluent hyper-reflective band in the subretinal space and infiltrates localized between the RPE and Bruch’s membrane. Active lesions can often present with outer retinal fuzzy borders, PED, and subretinal fluid, which resolve with treatment and may leave behind subretinal fibrosis, disruption of the outer retina, and retinal thinning.

FA and ICGA also provide additional structural information about disease processes associated with PVRL. While FA findings including optic disc leakage, retinal vascular leakage or petaloid edema may be observed in other endogenous uveitis syndromes, their presence should not exclude the possibility of PVRL. Moreover, ICGA can help to distinguish PVRL from other uveitides with more characteristic ICGA findings.

Given the advances in ultra-wide field imaging to document and follow lesions, complemented by OCT, fundus autofluorescence, and traditional angiography, multimodal imaging is a mainstay for the diagnosis and management of PVRL. There is a continued need for quantitative research using these methods, as well as functional assessments including ERG [35]. Applying a multimodal approach enhances the physician’s ability to accurately and promptly diagnose PVRL. Specifically, fundus photography allows for direct visualization of retinal and subretinal lesions as well as detection of subtle changes, particularly in the context of prior diffuse RPE changes. In addition, fundus autofluorescence and OCT provide meaningful structural information including outer retina, RPE, and sub-RPE lesions that may guide a clinician towards a definitive vitreous or retinal biopsy. Early diagnosis of PVRL not only hastens potentially vision-improving treatment but may also spur urgent investigation and management for primary CNS lymphoma, significantly improving patient health and quality of life.

## Data Availability

Data sharing is not applicable to this article as no datasets were generated or analyzed during the current study.

## Abbreviations

PVRL: Primary vitreoretinal lymphoma
FAF: Fundus autofluorescence
OCT: Optical coherence tomography
CNS: Central nervous system
RPE: Retinal pigment epithelium
VHRL: Vertical hyperreflective column
ICGA: Indocyanine green angiography
FA: Fluorescein angiography
ERG: Electroretinography
PED: Pigment epithelial detachment
CME: Cystoid macular edema
